# Short-term oleoyl-estrone treatment affects capacity to manage lipids in rat adipose tissue

**DOI:** 10.1186/1471-2164-8-292

**Published:** 2007-08-28

**Authors:** Anna Salas, Véronique Noé, Carlos J Ciudad, M Mar Romero, Xavier Remesar, Montserrat Esteve

**Affiliations:** 1 Departament de Nutrició i Bromatologia, Facultat de Biologia, Universitat de Barcelona, Av. Diagonal 645, 08028 Barcelona, Spain; 2 Departament de Bioquímica i Biologia Molecular, Facultat de Farmàcia, Universitat de Barcelona, Joan XXIII s/n, 08028 Barcelona, Spain; 3 CIBER Fisiopatología de la Obesidad y Nutrición (CB06/03), Instituto de Salud Carlos III, Spain

## Abstract

**Background:**

Short-term OE (oleoyl-estrone) treatment causes significant decreases in rat weight mainly due to adipose tissue loss. The aim of this work was to determine if OE treatment affects the expression of genes that regulate lipid metabolism in white adipose tissue.

**Results:**

Gene expression in adipose tissue from female treated rats (48 hours) was analysed by hybridization to cDNA arrays and levels of specific mRNAs were determined by real-time PCR. Treatment with OE decreased the expression of 232 genes and up-regulated 75 other genes in mesenteric white adipose tissue. The use of real-time PCR validate that, in mesenteric white adipose tissue, mRNA levels for Lipoprotein Lipase (LPL) were decreased by 52%, those of Fatty Acid Synthase (FAS) by 95%, those of Hormone Sensible Lipase (HSL) by 32%, those of Acetyl CoA Carboxylase (ACC) by 92%, those of Carnitine Palmitoyltransferase 1b (CPT1b) by 45%, and those of Fatty Acid Transport Protein 1 (FATP1) and Adipocyte Fatty Acid Binding Protein (FABP4) by 52% and 49%, respectively. Conversely, Tumour Necrosis Factor (TNFα) values showed overexpression (198%).

**Conclusion:**

Short-term treatment with OE affects adipose tissue capacity to extract fatty acids from lipoproteins and to deal with fatty acid transport and metabolism.

## Background

Experimental administration of Oleoyl-estrone (OE) induces the selective loss of body fat, without concurrent loss of body protein [1,2]. Since OE is synthesized from estrone by adipose cells [3] and released into the bloodstream, where its concentrations correlates with body fat mass [4,5], OE has been postulated as a lipostatic signal regulating body fat mass. The short-term effects of OE treatment in rats, involve the decrease in food intake, a decrease in body weight and an impressive decrease of cholesterol levels, mainly due to the sharp decrease of HDL-cholesterol that results in an increased cholesterol turnover rate [6]. This pattern has been reproduced also in obese humans [7]. The selective fall in total fat content is a direct consequence of the generalized decrease of adipose tissue mass in several locations [8].

The intra-venous administration of pharmacological doses of oleoyl-estrone causes mild estrogenic effects, and results in high circulating levels of estrone [9]. Oral administration of the estrone ester, however, prevents these effects by maintaining low plasma levels of both estrone and estradiol. Furthermore, oral OE treatment does not increase the total body estrogen content of the rat [10]. The lack of noticeable estrogenic effects has been confirmed when an obese man used OE treatment for lose weight without secondary effects [7]. At the moment, the mechanism underlying oleoyl-estrone action remains unknown. In this respect, the involvement of receptors different from classic estrogen receptors has been proposed [9], although it seems that the mechanism involves pathways other than those activated by forced food restriction, as pair-fed models [11].

It seems evident, however, that the OE action must be related with the ability of white adipose tissue to manage lipidic compounds. For this reason, the objective of the present work was to determine if a short-term OE treatment, in spite of its effects on food intake, caused significant changes in the expression of genes that regulate lipid metabolism in white adipose tissue.

## Results

Wistar rats were subjected to an oral treatment with oleoyl-estrone. After 48 hours, their body weight and food intake were determined as well as the levels of different metabolites in serum (Table 1). Treated animals showed significant lower percentages of body weight variation than controls, together with a noticeable decrease in food intake. Plasmatic levels of metabolites did not present important changes in treated animals, except for cholesterol and HDL-cholesterol that showed significant decreases compared to the controls.

**Table 1:** Weight change, food intake and plasma parameters of Wistar rats at 48-hours after the onset of an oral treatment with oleoyl-estrone (OE)

Parameter	Group	Mean ± SEM
Body weight (% of initial)	Control	101 ± 0.89
	OE	96.8 ± 0.76 *
Cumulative food intake (g)	Control	42.9 ± 2.11
	OE	29.7 ± 2.61 *
Glucose (mM)	Control	7.72 ± 0.20
	OE	7.65 ± 0.31
Total Cholesterol (mM)	Control	1.48 ± 0.13
	OE	0.88 ± 0.04 *
HDL-cholesterol (mM)	Control	0.98 ± 0.14
	OE	0.22 ± 0.02 *
HDL/total cholesterol ratio (%)	Control	66.2 ± 7.21
	OE	23.7 ± 2.05 *
Non-esterified fatty acids (mM)	Control	0.56 ± 0.08
	OE	0.60 ± 0.04
3-hydroxybutyrate (mM)	Control	0.28 ± 0.02
	OE	0.30 ± 0.06
Triacylglycerols (mM)	Control	0.91 ± 0.09
	OE	0.79 ± 0.10

The data are the mean ± SEM of data from six animals per group. Statistical comparison between groups was determined using the Student's *t *test. * = P < 0.05

The expression profile of the 1168 genes included in the specific Atlas Rat Array 1.2 (Clontech) was analyzed in the mesenteric white adipose tissue from control animals and treated with oleoyl-estrone. Treatment with OE decreased the expression of 232 genes and up-regulated 75 other genes in mesenteric white adipose tissue. Table 2 shows 18 down-regulated and 7 up-regulated selected genes that expressed a ratio lower than 0.5 or higher than 1.50 when compared with control group in mesenteric white adipose tissue. FAS, ACC, FABP4, LPL, CPT1b, HSL and FATP1 showed ratios lower than 0.40. The differentially expressed genes were classified according to their function in several categories and their role in the intermediate metabolism, as Complex Lipid Metabolism, Simple Lipid Metabolism, Other Traffiking & Targeting Proteins, etc. These data are deposited in Gene Expression Omnibus (GSE8538).

**Table 2:** Genes that are differentially expressed in mesenteric white adipose tissue of OE-treated rats

	Ratio	GeneBank	Functional Classification
**Down-regulated genes**			

Fatty acid synthase (FAS)	0.11	M76767	Metabolism of cofactors, vitamins & related substances
Acetyl-CoA carboxylase (ACC)	0.19	J03808	Simple Lipid metabolism
Prostaglandin d2 receptor	0.30	U92289	Hormone Receptors
Fatty acid transport protein (FATP1)	0.31	U89529	Other membrane channels & Transporters
Very long chain acyl-coa dehydrogenase (vlcad)	0.32	D30647	Metabolism of Cofactors, Vitamins & Related Substances
Lipoprotein lipase (LPL)	0.33	L03294	Complex Lipid Metabolism
Slow voltage-gated potassium channel protein	0.33	M22412	Voltage-gated ion channels
Low density lipoprotein receptor	0.35	X13722	Other receptors
Pyruvate dehydrogenase kinase kinase	0.37	L22294	Intracellular kinase network members
Adipocyte fatty acid-binding protein (FABP4)	0.38	U75581	Other Trafficking & Targeting proteins
Carnitine palmitoyltransferase 1b (CPT1b)	0.39	D43632	Simple Lipid metabolism
Hormone-sensitive lipase (HSL)	0.40	U40001	Simple Lipid Metabolism
Cytochrome P450 2c7	0.42	M18335	Complex Lipid Metabolism
Glucose-6-P-dehydrogenase	0.45	X07467	Complex Carbohydrate Metabolism
Retinoid X receptor beta	0.45	M81766	Nuclear receptors
Brain fatty acid binding protein	0.45	U02096	Other Trafficking & Targeting proteins
Adenyl cyclase	0.46	M80633	Adenylate/Guanylate Cyclases & Diesterases
Proteasome 26s subunit 2	0.47	D50694	Proteosomal Proteins

**Up-regulated genes**			

Insulin receptor-related receptor alpha subunit	1.52	M90661	Intracellular Kinase network members
Cytochrome p450 4f5	1.72	U39207	Complex Lipid Metabolism
Tumor necrosis factor alpha (TNFα)	1.77	X66539	Growth Factors, Cytokines hemokines
GTP-binding protein	1.82	L19699	G Proteins
Ras-gtpase activation protein	1.85	L13151	GTP/GDP exchangers & GTPse activity modulators
Cytochrome p450 4b1	2.01	M29853	Other Metabolism Enzymes
Protein arginine n-methyltransferase 1	3.59	U60882	Intracellular Adaptors eceptor Associated Proteins

The expression of each gene is reported as the ratio of the value obtained after each treatment relative to control after normalization of the data.

Next, we proceeded to validate the differential expression of specific outlier genes to verify the changes in mRNA levels. Real-time PCR offers a non hybridization-based detection and was chosen as complementary to arrays. The selected genes were analyzed by real-time PCR under the same experimental conditions as for the Array analyses.

**Figure 1:**
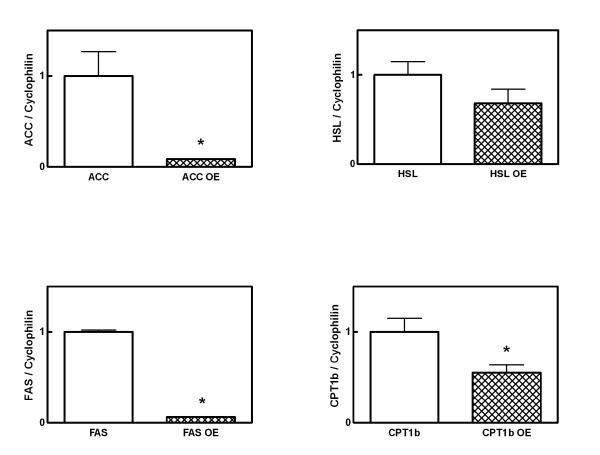
Quantization of HSL, ACC, FAS, and CPT1b by real-time PCR in samples of mesenteric white adipose tissue of treated rats during 48-hours with OE. The results express the levels of mRNA, corrected by cyclophilin values and expressed as a percentage of controls. Results express the means ± SEM of 4 different experiments. Asterisks indicate differences from controls: * P < 0.05.

**Figure 2:**
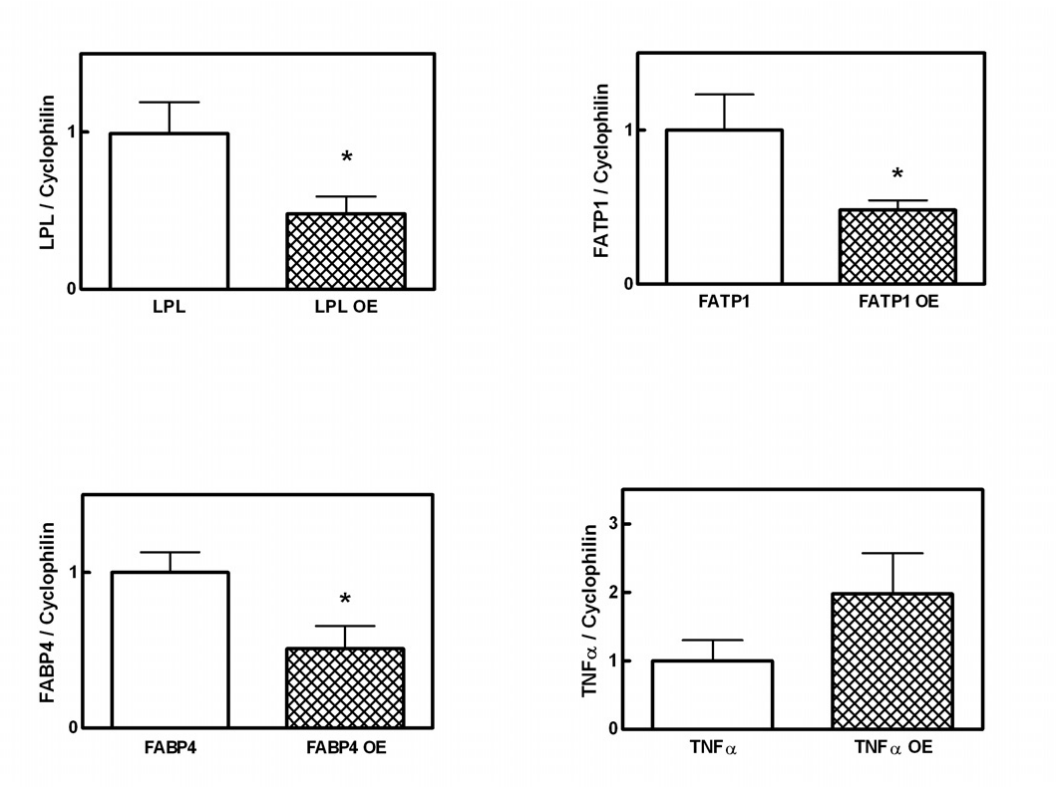
Quantization of LPL, FABP4, FATP1 and TNFα by real-time PCR in samples of mesenteric white adipose tissue of treated rats during 48-hours with OE. The results express the levels of mRNA, corrected by cyclophilin values and expressed as a percentage of controls. Results express the means ± SEM of 4 different experiments. Asterisks indicate differences from controls: * P < 0.05.

In mesenteric white adipose tissue (Figure 1), it was determined that mRNA levels for HSL were decreased by 32%, whereas those of FAS, ACC and CPT1b were decreased by a 95%, 92% and 45% respectively upon treatment with OE. In the Figure 2 can be seen that treatment with OE decreases mRNA expression of FATP1 by 52%, FABP4 by 49% and LPL by 52%. Conversely, TNFα showed overexpression (198%) as a consequence of OE-treatment. All these changes were statistically significant (p < 0.05), except for HSL and TNFα. These results confirmed the RNA data obtained in the screening performed using the cDNA arrays.

## Discussion

The application of genomic technologies to study different metabolic profiles has been widely used [12-14], especially in the case of adipose tissue or obesity. The application of this miniaturized hybridization technology allows the simultaneous analysis of the expression of hundreds of genes and pride the opportunity to discover abnormalities in the expression of many genes. In this case, the experience has been positive, since we have detected many genes underexpressed, and a more reduced amount of overexpressed genes, as a consequence of OE treatment. A minimal part of this abundant information has been shown in the Table 2, and we have only used which referred to lipid metabolism.

The short-term treatment with OE reproduced the pattern previously described [6], where the decrease caused in body weight, food intake and in HDL-cholesterol levels were the main traits. Under these standard conditions, we were able to determine the gene expression profile in tissue samples extracted from OE-treated animals and confirm by real-time PCR the changes in the expression of some specific genes. In the mesenteric white adipose tissue were selected different enzymes implied either in storage or in the mobilization of lipids; LPL as a main inductor of fatty acid uptake from lipoproteins; FATP1 and FABP4 implied in fatty acid transport across the membrane and citosol respectively; ACC and FAS as regulatory enzymes in the synthesis of fatty acids; HSL as main responsible for triacylglycerol mobilization and CPT1b as a regulatory pathway for beta-oxidation. Furthermore, TNFα, a regulatory peptide that exerts an important control on tissue proliferation and the uptake and storage of metabolites was also selected.

Lipoprotein lipase (LPL) is the key enzyme for the hydrolysis of circulating triglycerides (in chilomicra and VLDL lipoproteins) into free fatty acids and glycerol and plays a major role in adipocyte lipid storage and hence the regulation of obesity [15]. LPL is thought to act as a "gate keeper" for fatty acid uptake into organs [16], in spite that other pathways must be implied, because both humans and mice without adipose tissue LPL, still have some essential fatty acids in adipose tissue [17]. The under expression of LPL by OE-treatment is consistent with the previously described loss of weight, that is a consequence of decrease in adipose tissue weight [8]. Thus, if adipose tissue has lost some capacity to extract lipid from circulating lipoproteins, a decrease in the accumulation of lipids will be the immediate consequence. An inhibitory action of estrogens on lipoprotein lipase has been described to explain the inhibition of obesity development by estrogen replacement therapy in rats [15]; however, we do not have any evidence that support a similar action for the inhibitory effect of OE treatment. Furthermore, the underexpression of LPL could be also influenced by TNFα overexpression [18], due to its inhibitory action on LPL expression.

The decreased expression of fatty acid transport proteins (FATP1 and FABP4) confirms the previous statement, and then a low capacity to release fatty acids from lipoproteins is completed with a decreased transport into the cell (low FATP1 expression), that is accordance with the strength of their expression and the appearance of obesity [19]. Furthermore, the decrease in FABP4 expression is in accordance with a decrease in fatty acid utilization, and may be a consequence of the decreased food intake, as FABP4 seems to be up regulated by diet [20].

Lipogenesis implies the synthesis of fatty acids from non lipid substrates and is played in liver and adipose tissue and is under control of hormones and metabolites from dietary origin. Thus, insulin and glucose activate the lipogenic pathway, and conversely, glucagon and poly-unsaturated fatty acids inhibits this process [21]. These regulatory actions may imply the action of different transcription factors and nuclear receptors, in spite of the noticeable differences between human and rodent adipose tissues [22]. Fatty acid synthase activity and their mRNA expression are usually taken as markers of lipogenesis [23], since the alterations in activity are due primarily to alterations in the transcription rate. A sharp decrease in the expression of FAS, as in OE-treated rats, will imply an important decrease in its activity, and then a decrease in the synthesis *de novo *of fatty acids in adipose tissue. Moreover, the decrease in the expression of ACC may act reinforcing the slowing of fatty acid synthesis pathway. However, it must be taken into account the dual action of ACC, since the increase of their activity can act as a precursor of malonyl CoA as an inhibitor of mitochondrial translocation of fatty acids to beta-oxidation [24].

The decreased capacity to catabolize fatty acid can be a consequence both of a decrease in adipose tissue ability to degrade triacylglycerols and a decrease in the beta-oxidation pathway. Thus, a clear tendency to decrease in the expression of HSL, as HSL activity regulates lipolytic pathway [25], must be interpreted as adipose tissue reduces the hormone-dependent triacylglycerol degrading capacity. Furthermore, the passage of fatty acids to mitochondria is compromised as the expression of carnitine palmitoyltransferase 1b (CPT1b) shows a low expression level. Since CPT1b is the isoform present in adipose tissue [26], regulates the entrance to oxidation pathway, in spite of the low activity attributed to white adipose tissue [27], its low expression levels emphasizes the general trend to show low fatty acid oxidation rates as a consequence of OE-treatment. Moreover, this low pace activity can be reinforced by the inhibition caused by malonyl-CoA, that could be accumulated as a consequence of decreased FAS expression, since its decrease is faster than that of ACC (the expression of ACC has been reduced to 0.47 with 24-hour treatment, whereas FAS expression was reduced to 0.17 (data not shown).

Tumour necrosis factor-α (TNFα) can act both as autocrine or a paracrine factor, playing a central role in the control of production of several cytokines or acute phase proteins [28]. TNFα synthesis in adipose tissue occurs in stromal vascular and matrix fraction including macrophages. TNFα plays a main role in the inflammatory state that is characteristic of obesity, and the increase in its levels may be related with the inhibitory effect on insulin receptor signalling pathway, the diminishing triacylglycerol depots in adipose tissue or in the development of apoptosis [28]. Thus the tendency to increase the expression of TNFα could strengthen different mechanisms that will achieve with the decrease of lipidic depots in white adipose tissue, inhibiting the action of insulin and promoting the apoptosis, in spite of their inability to promote an increased HSL response. The different sensitivity to OE treatment in different adipose tissue sites [29], and the selectively different response of OE treatment in front of a single food deprivation [11], must be taken into account to infer that this treatment induces a tendency to increase the TNFα expression that can collaborate with the global process of lipid mobilization, probably through the stimulation apoptotic pathways, since a generalized increase in apoptosis markers has been described as a consequence of OE-treatment [29].

## Conclusion

Short-term treatment with OE affects the capacity of adipose tissue to extract fatty acids from lipoproteins (decrease in LPL expression), to transport fatty acids (decreases in FABP and FATP), to generate fatty acids from precursors (decrease in ACC and FAS expression) and to catabolize their fatty acids (decreases in CPT1b expressions), where the global process is controlled by regulator factors such as TNFα. This impaired ability to obtain metabolites from food, and the restricted ability to store them in adipose tissue are key observations of the initial tracks to establish the mechanism of action of OE.

## Methods

### Animals

Female Wistar rats (Harlan-Interfauna Ibérica, Sant Feliu de Codines, Spain) weighing initially 220–230 g were housed in individual cages under a light cycle (on from 08.00 to 20.00) and in a temperature-controlled environment (20–22°C). Food (standard rat chow pellets, from Panlab, Barcelona, Spain) (available energy: 13.6 MJ/kg) and water were provided *ad libitum*.

All procedures were in accordance with the guidelines for the use of experimental animals established by the EU, Spain and Catalonia, and were approved by the Animal Handling Ethics Committee of the University of Barcelona.

### Treatment

The rats were given a daily oral gavage of 10 μmol/kg/day of oleoyl-estrone (OED, Barcelona, Spain) in 0.2 ml of sunflower oil during two days, by means of stomach cannulae. Controls received only the oil gavage.

### Tissue samples and plasma determinations

After 48 hours of the beginning of treatment, fed rats were killed by decapitation and blood was recovered in plastic beakers. Serum was separated and frozen for analysis. The mesenteric white adipose tissue was also dissected and frozen in liquid nitrogen and stored at -80°C. This adipose location was used as showed the higher sensitivity to OE treatment [29].

The serum was later used for the measurement of total cholesterol (Menarini, Firenze, Italy), HDL-cholesterol using the same kit after using the HDL-cholesterol precipitant rat reagent (Randox, Crumlin, UK), glucose (Sigma, St. Louis MO, USA), non-esterified fatty acids (Wako, Richmond VA, USA), 3-hydroxybutyrate (Roche, Mannheim, Germany) and total triacylglycerols (Biosystems, Barcelona, Spain).

### RNA isolation

Total RNA was extracted from adipose mesenteric tissue using Tripure isolation kit (Roche). RNA concentration and quality were measured by spectrophotometric analysis at 260 nm and 280 nm using Nanodrop equipment (Wilmington DE, USA). RNA integrity was assessed by electrophoresis in agarose gel.

### cDNA arrays

Gene expression was analysed by hybridization to cDNA arrays (Atlas™ Rat Array 1.2 from BD-Clontech, Mountain View CA, USA). We selected this array because it was specific for rat and included genes implied in multiple metabolic actions, fact that was appropriate for an initial screening of OE action. Radiolabeled cDNA probes were prepared from 5 μg of total RNA. Briefly, the RNA was incubated for 2 min at 70°C followed by 2 min at 50°C with 1 μl of the primer mix, containing the 1168 primers for the genes presented in the array. The RT reaction was carried out using 100 U MMLV-RT, 40 U RNAsin (Promega, Madison WI, USA) and 35 μCi [α-^33^P] dATP (Amersham Biosciences, Piscataway NJ, UK) for 25 min at 50°C. Labeled cDNA was purified from unincorporated nucleotide with Atlas NucleoSpin^® ^Extraction Kit (Clontech). Nylon membranes were pre-hybridized in ExpressHyb™ (Clontech) with 100 μg/ml DNA salmon sperm for 30 min in an oven at 68°C. Then, the ^33^P-labeled probe was added and hybridization continued overnight at the same conditions. Afterwards, membranes were washed lowering the astringency progressively to 0.1 × SSC, 0.5% SDS at 68°C and placed in contact with europium screens (Kodak, Rochester NY, USA) for 15 days. The screens were scanned with a Storm 840 phosphorimager (Molecular Dynamics, Sunnyvale CA, USA).

### Array data analysis

Image analysis and quantification were carried out with Atlas Image 2.01 software (Clontech). After grid assignment, the adjusted intensity for each gene was calculated by subtracting the background. This value was used as the input for the GeneSpring 6.1 program (Agilent, Palo Alto CA, USA), that allows normalization of data analysis from different experiments, the generation of restriction lists, and the functional classification of the differentially expressed genes. Normalization was applied in two steps: (i) 'per chip normalization', where each data point was divided by the 50^th ^percentile of all measurements in its array and (ii) normalization of each sample against the median of the control samples. The expression of each gene is reported as the ratio of the value obtained after each treatment relative to control after normalization of the data. Then data were filtered using the control strength, a control value calculated using the Cross-Gene Error Model based on replicates. Measurements with higher control strength are relatively more precise than measurements with lower control strength. Genes that didn't reach this value were discarded. Lists of differentially expressed genes considering a 1,5 or 0.5 fold expression were generated using data from 2 animals for each condition. These cutoffs of 1.5 or 0.5 were chosen given that small changes in gene expression may represent important changes downstream of those differentially expressed genes. The genes in these lists were further classified according to their function.

### Real-time PCR

The selected targets for real-time PCR were LPL, FAS, HSL, FABP4, FATP1, ACC, CPT1b and TNF-α. Specific mRNA levels were determined by semi-quantitative real-time PCR. To disrupt the potential formation of secondary structures, 2 μg total RNA and 40 μg oligo dT primers (Roche) in 10 μl were incubated for 5 min at 70°C and then chilled on ice. Complementary cDNA was synthesized by adding to the mixture 200 U MMLV RT (Promega), 25 U RNAsin (Promega), 0.5 mM dNTPs (GeneCraft, Lüdinghausen, Germany) and MMLV RT buffer. Twenty-five μl of reaction mixture was incubated at 42°C for 60 min, with the cDNA product used for subsequent PCR amplification with specific primers.

The real-time assay was performed using an iCycler iQ (Bio-Rad, Hercules CA, USA). Rat FAS, TNFα, ACC, FABP4, HSL, CPT1b and FATP1 mRNA expressions were analyzed using SYBR Green and LPL and cyclophilin using Taq Man methodology. The signals corresponding to the cyclophilin mRNA were used to normalize the changes in mRNA levels for each particular case.

Primers from genes analyzed by SYBR Green, were designed (Primer 3 program) to be exon-spanning to avoid amplification of genomic DNA. To confirm the amplicon length PCR products were resolved on 2% agarose gel. Forward and reverse primers sequences for PCR amplification and the length of the PCR product were:

FAS 5'-CTTGGGTGCCGATTACAACC-3' and 5'-GCCCTCCCGTACACTCACTC-3' (185 bp);

TNFα 5'-GGCTCCCTCTCATCAGTTCCA-3' and 5'-GCTTGGTGGTTTGCTACGA-3' (104 bp);

ACC 5'-AGGAAGATGGTGTCCCGCTCTG-3' and 5'-GGGGAGATGTGCTGGGTCAT-3' (145 bp);

FABP4 5'-CCTTTGTGGGGACCTGGAAA-3' and 5'-TGACCGGATGACGACCAAGT-3' (152 bp);

HSL 5'-CCCATAAGACCCCATTGCCTG-3' and 5'-CTGCCTCAGACACACTCCTG-3' (93 bp);

CPT1b 5'-GTGCTGGAGGTGGCTTTGGT-3' and 5'-TGCTTGACGGATGTGGTTCC-3' (152 bp);

FATP1 5'-GTGCGACAGATTGGCGAGTT-3' and 5'-GCGTGAGGATACGGCTGTTG-3' (106 bp).

The PCR mixture (20 μl final volumes) contained 10 ng cDNA, 1× iQ SYBR Green Supermix (Bio Rad) and 300 nM of forward and reverse primers.

Taq Man primers and probe for LPL (assay ID: Rn 00561482_ml) and cyclophilin (assay ID: Rn 00690933_ml) were assay-on-demand (AoD) gene expression products selected (Applied Biosystems, Foster City CA, USA). The PCR mixture (20 μl final volume) contained 10 ng cDNA, 1× iQ Supermix (Bio Rad) and 1× AoD mixture.

The thermal conditions were: initial denaturation of cDNA (3 min at 95°C), amplification of target cDNA (denaturation 30 s at 95°C and 40 cycles of amplification 1 min 60°C). Melting curve analysis for product identification and to discard dimmers presence was performed in the SYBER Green assay.

### Statistical methods

Array data analyses were conducted via a parametric comparison using all available error estimates as a filter based on variances calculated by the Cross-gene Error Model in GeneSpring 6.1 software (Silicon Genetics, Redwood City CA, USA). The Cross-gene Error Model performs a variance components analysis for the accurate comparison of mean expression levels between experimental conditions. In this model, separate estimates for two different kinds of random variation are used to estimate the variability in gene expression measurements: i) measurement variation: this comprises the lowest level of variation, corresponding to the variation of gene measurement of a single chip based on the actual value that would be obtained from a perfect measurement of the gene expression level for that sample, and ii) sample-to-sample variation: the variation between samples under the same condition reflecting biological or sampling variability.

For real-time PCR analyses, values are expressed as the mean ± S.E.M. Data were evaluated using the unpaired Student's *t*-test and analysis was performed with Prism 4.0 software (GraphPad Software, San Diego CA, USA). Differences in P-values < 0.05 were considered significant.

## Authors' contributions

AS took part in all parts of the study. VN and CJC contributed to design and to analyse the data obtained in the array essay. MMR contributed to carry out the RT-PCR. XR contributed to experimental design, animal managing and drafted the manuscript. ME supervised the study design and contributed to writing the manuscript. All authors made contributions to the final version of manuscript. All authors read and approved the final manuscript.
